# A Specialized Multinutrient Formula Attenuates High-Fat-Diet-Associated Muscle Dysfunction and Sarcopenic Obesity-Related Features in Adult Mice

**DOI:** 10.3390/nu18172886

**Published:** 2026-09-03

**Authors:** Hang Wang, Yi-Jing Guo, Shang-Ta Wang

**Affiliations:** 1 Department of Nutrition, Hungkuang University, Taichung 433304, Taiwan; yijing01520@gmail.com; 2 Institute of Food Safety and Risk Management, National Taiwan Ocean University, Keelung 202301, Taiwan; wst@mail.ntou.edu.tw

**Keywords:** sarcopenia, high-fat diet, skeletal muscle, nutritional supplementation, whey protein, inflammation, muscle function

## Abstract

**Background/Objectives**: Sarcopenia is an age-associated skeletal muscle disorder that may be aggravated by a high-fat diet (HFD). Given the multifactorial pathogenesis of sarcopenia, single-nutrient interventions may be insufficient. This study investigated whether a specialized multinutrient formula could attenuate muscle dysfunction and intramuscular lipid accumulation in adult mice under standard-diet and HFD-fed conditions. **Methods**: Male C57BL/6J mice were assigned to five groups: young control (YC), adult control (OC), adult control supplemented with the nutritional formula (OCN), adult HFD-fed mice (OH), and adult HFD-fed mice supplemented with the formula (OHN). A specialized multinutrient formula containing enriched whey protein, β-glucan, antioxidant vitamins, and essential trace minerals was orally administered five times per week for 12 weeks. Body composition, muscle histology, grip strength, intramuscular lipid accumulation, inflammatory gene expression, and protein markers related to caspase-associated apoptosis, proteolysis-related signaling, and tissue remodeling were assessed. **Results**: Compared with YC mice, adult control and HFD-fed mice exhibited reduced muscle strength and fiber cross-sectional area, increased adiposity, intramuscular lipid deposition, elevated TNF-α expression, and alterations in selected protein markers related to caspase-associated apoptosis, proteolysis-related signaling, and tissue remodeling. Nutritional supplementation improved muscle strength and fiber morphology, reduced intramuscular lipid deposition, lowered TNF-α expression, reduced cleaved caspase marker abundance, decreased p38 MAPK and MuRF-1 expression, and increased collagen type III expression. **Conclusions**: HFD aggravated muscle dysfunction and sarcopenic obesity-related features in adult mice, as evidenced by impaired muscle function, reduced muscle fiber cross-sectional area, increased intramuscular lipid accumulation, and changes in selected inflammatory, cleaved caspase-, protein turnover-related, and remodeling markers. Nutritional supplementation mitigated these alterations and improved muscle structure and function.

## 1. Introduction

With progressive aging of the global population, declines in skeletal muscle mass and function—a condition known as sarcopenia—have become increasingly prevalent [1]. Muscle quality is reported to decrease by approximately 1–2% per year with advancing age [2]. Other age-related alterations in body composition include increases in fat mass and decreases in skeletal muscle mass, even when body mass index (BMI) remains unchanged [3]. According to data from the Asian Working Group for Sarcopenia (AWGS), the prevalence of sarcopenia among older adults in Asia is approximately 5.5–25.7% [4]. Among Taiwanese adults aged 65 years and older, the prevalence of sarcopenia has been reported to be 23.6% in men and 18.6% in women [5].

Although sarcopenia is typically observed in older populations, it may manifest in younger individuals under certain conditions. Sarcopenia is also associated with prolonged physical inactivity, such as extended bed rest or sedentary lifestyles, severe malnutrition or eating disorders, and chronic diseases or inflammation that impair muscle quality and function [6,7,8]. Individuals affected by sarcopenia experience a higher risk of falls or injuries, reduced quality of life, increased disease burden, and elevated mortality risk [8]. The pathogenesis of sarcopenia is complex and involves both intrinsic factors, such as chronic low-grade inflammation, dysregulated autophagy, mitochondrial dysfunction, and neuromuscular degeneration, and extrinsic factors, such as inadequate nutrition, physical inactivity, and hormonal imbalance [2,9]. Notably, the relative contribution of these pathways may vary across individuals and disease stages. While fat redistribution is a well-documented phenomenon in aging populations—where visceral adipose tissue and intramuscular fat accumulation increase between ages 60 and 75 years while subcutaneous fat decreases, exacerbating muscle quality deterioration [10]—recent evidence suggests that similar unfavorable patterns can already impact younger individuals. In healthy young adults, early accumulation of visceral adipose tissue, despite a higher baseline proportion of subcutaneous fat, has been significantly associated with sub-clinical systemic inflammation, which subsequently impairs muscle quality and functional capacity at an earlier stage of life [11].

Sarcopenia frequently coexists with obesity, giving rise to a condition known as sarcopenic obesity. A recent report estimated the global prevalence of sarcopenic obesity among older adults as approximately 11% [12]. In response, the European Society for Clinical Nutrition and Metabolism (ESPEN) and European Association for the Study of Obesity (EASO) jointly proposed consensus diagnostic criteria defining sarcopenic obesity as the coexistence of excessive adiposity and impaired skeletal muscle quality or function [2,13,14]. As a result, sarcopenic obesity is now recognized as a high-risk geriatric syndrome. In the context of obesity, several pathological processes are thought to contribute to sarcopenia. For example, muscle progenitor cells may undergo adipogenic differentiation that leads to increased lipid infiltration within skeletal muscle. This process establishes a vicious cycle in which muscle loss and lipid accumulation synergistically accelerate functional decline [15,16]. Previous studies have demonstrated that both aging- and obesity-induced muscle loss are strongly associated with chronic inflammation, including activation of p38 mitogen-activated protein kinase signaling and increased expression of pro-inflammatory cytokines like tumor necrosis factor-α (TNF-α) and interleukin-6 (IL-6) [17,18].

Skeletal muscle quality is tightly regulated by the dynamic balance between muscle protein synthesis and muscle protein degradation. Disruption of this balance that favors protein degradation (synthesis < degradation) can lead to muscle atrophy and functional decline. The ubiquitin–proteasome system (UPS) is a major pathway involved in muscle protein degradation in which muscle-specific E3 ubiquitin ligases, such as muscle RING finger-1 (MuRF-1) and muscle atrophy F-box (MAFbx/Atrogin-1), act as key regulators of proteolysis [19,20]. However, because previous studies have shown that MuRF-1 and MAFbx expression are not always markedly elevated in high-fat-diet-induced muscle atrophy models, mechanisms beyond UPS-mediated proteolysis may also contribute to muscle loss [21]. Autophagy–lysosome pathways and apoptosis have therefore been proposed as alternative or complementary mechanisms involved in the progression of sarcopenia [22].

With increasing age, impaired immune function and dysregulation of autophagy–lysosome pathways reduce the clearance of damaged cellular components, promoting mitochondrial dysfunction and the release of cytochrome c, which in turn activates caspase-dependent apoptotic signaling [23]. Activation of caspase-3 and related pathways has been implicated in accelerated muscle cell loss under both aging and metabolic stress conditions. Differences in findings across experimental studies may partly reflect variations in model systems, including naturally aged models and HFD-associated muscle dysfunction models. Collectively, these observations highlight sarcopenia as a multifactorial public health challenge and underscore the urgent need for effective nutritional strategies that simultaneously target multiple pathological pathways.

Because sarcopenia arises from multiple interacting biological processes, recent research has shifted toward multinutrient strategies rather than single-nutrient approaches. Maintenance of muscle mass and strength is a central therapeutic goal, and nutrients such as whey protein, branched-chain amino acids (BCAAs), glutamine, and L-carnitine can support muscle protein synthesis and functional performance [24,25]. Moreover, growing evidence suggests that naturally derived bioactive compounds, including dietary fiber, are associated with skeletal muscle strength, lean mass, and grip performance [26,27]. These findings indicate that muscle health is influenced by both anabolic signaling and inflammatory regulation.

β-glucan, a polysaccharide derived from yeast and cereal sources, has attracted interest for its immunomodulatory and metabolic effects. Polycan, a purified β-1,3/1,6-glucan extracted from *Aureobasidium pullulans*, exhibits a stable triple helix structure and interacts with gut-associated immune cells to promote anti-inflammatory responses. Because chronic low-grade inflammation is a hallmark of aging and obesity-associated sarcopenia, β-glucan supplementation has been investigated as a potential intervention. Experimental studies have shown that β-glucan can reduce pro-inflammatory cytokine production, improve endurance performance, and attenuate biochemical markers of muscle damage [28,29,30,31].

Whey protein is a rapidly digestible, leucine-rich protein source that effectively stimulates postprandial muscle protein synthesis and helps preserve lean mass particularly in older adults. Compared with other protein sources, whey protein has high bioavailability and contains bioactive fractions that may support antioxidant defense and metabolic regulation [32,33,34]. Adequate intake of high-quality protein with sufficient leucine is essential for activating anabolic signaling pathways in aging muscle, and whey protein supplementation is associated with improvements in body composition and metabolic health in overweight and obese populations [35,36,37].

BCAAs—particularly leucine—serve as both substrates for protein synthesis and nutrient signals that activate the Akt/mTOR pathway, suppress catabolic signaling through FoxO transcription factors, and promote mitochondrial biogenesis via peroxisome proliferator-activated receptor gamma coactivator-1α (PGC-1α) [38,39,40,41,42]. These mechanisms are especially relevant in aging and sedentary conditions where reduced anabolic responses and increased proteolysis contribute to progressive muscle loss.

Micronutrients, such as vitamins A, C, D, E, and K, play essential roles in muscle remodeling, antioxidant defense, and extracellular matrix maintenance. Availability of these micronutrients influences muscle satellite cell activation and differentiation, whereas inadequate micronutrient status in older adults has been associated with impaired muscle quality, increased oxidative stress, and reduced remodeling capacity [43,44,45,46,47,48,49]. Collectively, these findings support the value of a comprehensive nutritional strategy that targets inflammation, protein turnover, apoptosis, and tissue remodeling.

Accordingly, the present study investigated whether a specialized multinutrient formula—comprising BCAA-enriched whey protein, black yeast fermentation extract rich in β-glucan, and essential vitamins and minerals—could improve skeletal muscle function and muscle quality under adult standard-diet and HFD-fed conditions. In this study, 15-week-old mice were used as adult mice to evaluate muscle-related responses, while HFD feeding was used to induce obesity-associated metabolic stress and muscle dysfunction. We hypothesized that this multinutrient formula would attenuate HFD-associated muscle dysfunction and intramuscular lipid accumulation by improving muscle functional performance, muscle fiber morphology, inflammatory status, and selected protein markers related to caspase-associated apoptosis, proteolysis-related signaling, and tissue remodeling.

## 2. Materials and Methods

### 2.1. Animal Experiment

This study used male C57BL/6J mice. Mice aged 4 weeks and 15 weeks were obtained from the National Laboratory Animal Center, National Applied Research Laboratories (Taipei, Taiwan). The 15-week-old mice were used as adult mice to evaluate muscle-related outcomes under standard-diet and HFD-fed conditions. All mice were housed in a specific pathogen-free facility under controlled environmental conditions (22 ± 2 °C; 12 h light/dark cycle). All animal experiments were reviewed and approved by the Institutional Animal Care and Use Committee of Hungkuang University (approval number: HK-11303).

After a 1-week acclimation period, mice were randomized into five groups (*n* = 5 per group): young control (YC), adult control (OC), adult control supplemented with the nutritional formula (OCN), adult HFD-fed mice (OH), and adult HFD-fed mice supplemented with the formula (OHN). Male mice were used to minimize potential variability related to the estrous cycle in metabolic and muscle functional outcomes. Randomization was performed after acclimation, and initial body weight was considered to avoid marked baseline differences among adult groups. The YC, OC, and OCN groups were fed a standard chow diet (LabDiet 5001), whereas the OH and OHN groups were fed a high-fat diet (TestDiet 58Y1). All mice had ad libitum access to food and water. The available nutritional compositions of the diets and specialized multinutrient formula are provided in Supplementary Table S1.

**Figure 1:**
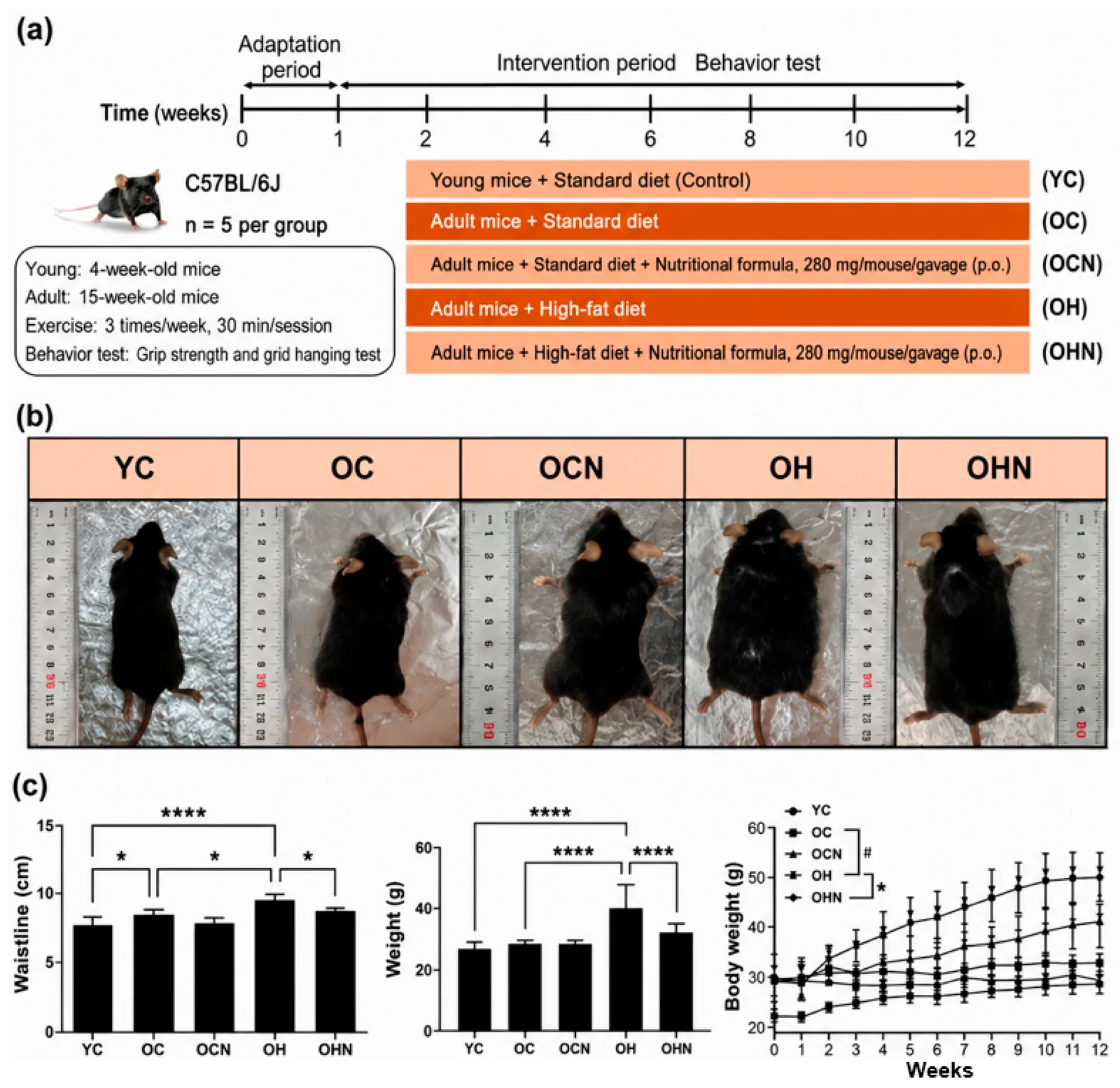
Effects of high-fat-diet feeding and nutritional supplementation on body morphology and body weight over the 12-week experimental period. (**a**) Schematic of the experimental design. After a 1-week acclimation period, OCN and OHN group mice were orally administered the specialized multinutrient formula five times per week for 12 weeks. Non-supplemented mice received purified water by oral gavage on the same schedule. All mice underwent the same non-loaded ladder-climbing activity throughout the experimental period. Grip strength was assessed at weeks 2, 4, 6, 8, 10, and 12, and the grid hanging test was performed at week 12. (**b**) Representative images of body morphology among the different groups at week 12. (**c**) Body weight changes during the 12-week experimental period. Data are presented as the mean ± SD; *n* = 5 mice/group. Longitudinal body weight data were analyzed using a mixed-effects model. In panel (**c**), # indicates a significant difference between the OC and OH groups at week 12 (*p* < 0.05). * *p* < 0.05 and **** *p* < 0.0001. YC, young control; OC, adult control; OCN, adult control with nutritional formula; OH, adult high-fat diet; OHN, adult high-fat diet with nutritional formula.

The overall study design is presented in Figure 1a. Mice in the OCN and OHN groups received the specialized multinutrient formula by oral gavage five times per week for 12 weeks. Immediately before each administration, 280 mg of the powdered formula was dissolved in 400 μL of purified water. Each supplemented mouse received a fixed dose of 280 mg per gavage, irrespective of body weight. Non-supplemented mice received an equivalent volume of purified water by oral gavage on the same schedule. Because a fixed dose was administered, the body-weight-adjusted exposure varied according to body weight during the experimental period. The approximate exposure range was 14.0 g/kg for a 20 g mouse, 9.3 g/kg for a 30 g mouse, 7.0 g/kg for a 40 g mouse, and 5.4 g/kg for a 52 g mouse. The final administration was performed one day before sacrifice.

Food intake was recorded weekly at the cage level during the experimental period and expressed as estimated g/mouse/day by dividing cage-level food consumption by the number of mice per cage. Each cage contained five mice. Total caloric exposure was estimated descriptively using the caloric densities of the standard chow diet, HFD, and specialized multinutrient formula. Because food intake was recorded at the cage level, these data were used for descriptive interpretation rather than individual mouse-level statistical analysis. Estimated food intake and caloric exposure are summarized in Supplementary Table S2.
**Component Category****Main Components Included** **in the Formula****Rationale for Inclusion**Protein sourceEnriched whey proteinTo provide high-quality protein to support muscle protein synthesisAmino acid profileBranched-chain amino acids, including leucine, isoleucine, and valineTo support anabolic signaling and muscle protein turnoverBioactive polysaccharideBlack yeast fermentation extract rich in β-glucanTo modulate inflammatory and metabolic responsesAntioxidant vitaminsVitamins A, C, D, E, and KTo support antioxidant defense, tissue remodeling, and extracellular matrix maintenanceEssential trace mineralsEssential minerals and trace elementsTo support metabolic regulation and skeletal muscle function

To minimize variability in baseline muscle activity, all mice underwent the same standardized non-loaded ladder-climbing activity throughout the 12-week experimental period. The protocol was performed three times per week, with each session lasting 30 min. During each session, mice repeatedly climbed the ladder without additional external loading. Exercise sessions were performed during the daytime according to facility operation and animal handling schedules. The same protocol was applied equally across all groups and was therefore considered a standardized background condition rather than an independent experimental intervention. Thus, the nutritional formula was evaluated under a standardized exercise background rather than under sedentary conditions.

Forelimb grip strength was assessed at weeks 2, 4, 6, 8, 10, and 12, whereas four-limb muscle endurance was assessed using the grid hanging test at week 12. Each mouse was tested five times per session. Mean values were calculated for each individual mouse and for each experimental group. All functional assessments were performed at the same time of day to minimize circadian variability, and mice were acclimated to the testing apparatus prior to data collection. Functional assessments were performed using standardized procedures, consistent timing, repeated measurements, and objective outcome recording to minimize measurement bias.

Body weight and waist circumference were measured weekly throughout the experimental period. At the end of the 12-week intervention, mice were euthanized and their body morphology was documented. Hindlimb skeletal muscles and adipose tissues were excised and weighed. Skeletal muscle tissues subsequently underwent histological and molecular analyses to evaluate muscle morphology, lipid accumulation, inflammatory gene expression, and selected protein markers related to caspase-associated apoptosis, protein turnover, and tissue remodeling.

### 2.2. Hematoxylin and Eosin (H&E) Staining

Gastrocnemius muscle tissues were fixed in 10% formalin, dehydrated, and embedded in paraffin. Subsequently, tissues were sectioned at a thickness of 5 μm, stained with H&E, and imaged using a light microscope. The diameter and cross-sectional area of muscle fibers were quantified using ImageJ software (version 1.54; National Institutes of Health, Bethesda, MD, USA). For qualitative presentation, one representative image was selected from each group. For quantitative CSA analysis, one image was analyzed for each mouse, and three mice were analyzed per group. In each image, 2–3 clearly identifiable muscle fibers were measured, and the mean value was calculated for each mouse. Each mouse, rather than each individual fiber, was treated as the biological experimental unit for statistical analysis.

### 2.3. Oil Red O Staining

Frozen sections of gastrocnemius muscle were fixed with 10% formalin and stained with filtered oil red O solution for 10–15 min. Excess background staining was removed by rinsing with 60% isopropanol. In some cases, hematoxylin counterstaining was performed to visualize cell nuclei. Sections were mounted using an aqueous mounting medium and observed under a microscope. Lipid droplets were visualized in red and nuclei were stained blue.

### 2.4. Grip Strength Test

Grip strength was measured every 2 weeks for each mouse using an electronic grip strength meter (REZ-5, Hong-Ming Technology Co., Ltd., New Taipei City, Taiwan). Prior to testing, mice were allowed to acclimate to the grid. During the test, mice grasped the horizontal metal bar connected to the force transducer and the tail was gently pulled backward in a horizontal direction until the forelimbs released the bar. The maximum grip force was automatically recorded. Each mouse was tested five times, and the average value was used as the grip strength for analysis.

### 2.5. Grid Hanging Test

The grid hanging test was performed at week 12 using a 40 × 40 cm wire grid to assess motor coordination and muscle endurance. Mice were allowed to adapt to the grid before testing. After the mouse grasped the grid, it was gently inverted 180°, allowing the mouse to hang freely until it fell into the cage below. The hanging time was recorded. Each test was repeated five times, and the average duration was calculated for analysis.

### 2.6. Western Blotting

Muscle tissues were lysed using RIPA buffer containing protease and phosphatase inhibitors. Protein concentrations were determined using the bicinchoninic acid (BCA) assay and normalized. Equal amounts of protein were separated by 12.5% sodium dodecyl sulfate-polyacrylamide gel electrophoresis and transferred onto polyvinylidene fluoride membranes. Membranes were blocked for 1 h and incubated overnight at 4 °C with the following primary antibodies: anti-cleaved caspase-3 (1:1000, #9664, Cell Signaling Technology, Danvers, MA, USA; expected molecular weight: 17/19 kDa), anti-cleaved caspase-8 (1:1000, #8592, Cell Signaling Technology; expected molecular weight: 18/43 kDa), anti-cleaved caspase-9 (1:1000, #9509, Cell Signaling Technology; expected molecular weight: 37 kDa), anti-Bcl-2 (1:1000, #633501, BioLegend, San Diego, CA, USA; expected molecular weight: 25 kDa), anti-Bax (1:1000, #ab32503, Abcam, Cambridge, UK; expected molecular weight: 21 kDa), anti-phospho-Akt (Ser473) (1:1000, #4058, Cell Signaling Technology; expected molecular weight: 60 kDa), anti-FoxO3a (1:1000, #bs-1548R, Bioss, Woburn, MA, USA; expected molecular weight: 74 kDa), anti-phospho-FoxO3a (Ser253) (1:1000, #bs-3140R, Bioss; expected molecular weight: 71 kDa), anti-p38 MAPK (1:1000, #14064-1-AP, Proteintech, Rosemont, IL, USA; expected molecular weight: 38–42 kDa), anti-MuRF-1/MuRF-2/MuRF-3 (1:1000, #ab172479, Abcam; expected MuRF-1 band: approximately 40 kDa), anti-collagen type III (1:1000, #22734-1-AP, Proteintech; calculated molecular weight: 139 kDa; observed molecular weight: approximately 140–180 kDa), anti-TGF-β (1:1000, #3711, Cell Signaling Technology; expected molecular weight: 25 kDa, with reported forms at 12 and 45–65 kDa), and anti-GAPDH (1:10,000, #GTX100118, GeneTex, Irvine, CA, USA; expected molecular weight: 36 kDa). HRP-linked goat anti-rabbit IgG (1:2000, #7074, Cell Signaling Technology) or HRP-linked horse anti-mouse IgG (1:2000, #7076, Cell Signaling Technology) secondary antibodies were used according to the host species of the primary antibodies. The resulting protein bands were detected using the LAS-4000 imaging system (FUJIFILM, Tokyo, Japan) and quantified using ImageJ software (version 1.54; National Institutes of Health, Bethesda, MD, USA).

### 2.7. Quantitative Real-Time PCR (qRT-PCR)

Total RNA was extracted from gastrocnemius muscle tissues using TRIzol™ reagent (Invitrogen, Thermo Fisher Scientific, Waltham, MA, USA). RNA was isolated using BCP and isopropanol, air-dried, and then dissolved in DEPC-treated water. The RNA solution was heated on a heat block and reverse-transcribed into complementary DNA (cDNA) using the HiSenScript™ RH(-) RT PreMix Kit (iNtRON Biotechnology, Seongnam, Republic of Korea) according to the manufacturer’s instructions. qRT-PCR was performed using specific forward and reverse primers for each target gene. The primer sequences were as follows:
**Gene****Primer Direction****Sequence (5′–3′)**Tnf-αForwardCTGTGAAGGGAATGGGTGTT
ReverseGGTCACTGTCCCAGCATCTTIl-6ForwardCCTCTGGTCTTCTGGAGTACC
ReverseACTCCTTCTGTGACTCCAGCGapdhForwardGCGACTTCAACAGCAACTC
ReverseGGTCCAGGGTTTCTTACTCC

The qPCR protocol was as follows: initial denaturation at 95 °C for 3 min, followed by 40 cycles of denaturation at 95 °C for 5 s, annealing at 60 °C for 1 min, and extension at 72 °C for 30 s. Relative gene expression levels were normalized to Gapdh and calculated using the 2^−ΔΔCt^ method. All samples were analyzed in technical triplicate. Technical triplicates were averaged for each mouse before analysis, and each mouse was treated as the biological experimental unit. Statistical testing was performed using animal-level ΔCt values, whereas relative expression values were used for data presentation.

### 2.8. Statistical Analysis

Statistical analyses were performed using GraphPad Prism software (version 10.1; GraphPad Software, Boston, MA, USA). All data are presented as the mean ± standard deviation (SD). Based on preliminary data, prior studies using similar animal models, and feasibility considerations, 5 mice per group were used in this study. Sample sizes for each analysis are indicated in the corresponding tables and figure legends. Longitudinal body weight and grip strength data were analyzed using a mixed-effects model with group, time, and group-by-time interaction as fixed effects, with individual mouse as the repeated biological unit. Data normality and homogeneity of variances were assessed using the Shapiro–Wilk and Brown–Forsythe tests, respectively, before ANOVA for all endpoint outcomes, including qRT-PCR ΔCt values. Endpoint data were analyzed using one-way analysis of variance (ANOVA), followed by Tukey’s multiple-comparison test where appropriate. qRT-PCR data were statistically analyzed using animal-level ΔCt values. Food intake and estimated caloric exposure were summarized descriptively because intake was recorded at the cage level. A *p*-value < 0.05 was considered statistically significant.

## 3. Results

### 3.1. Changes in Body Composition

Representative images of body morphology and body weight changes in the different groups at the end of the 12-week study period are shown in Figure 1b. Compared with the YC group, adult mice exhibited increased body size and adiposity, with the OH group displaying the most pronounced obese phenotype. Following supplementation with the specialized multinutrient formula, body weight and waist circumference were lower in the supplemented groups, particularly in the OHN group.

Relative to the YC group, waist circumference increased by approximately 10% in the OC group and 22% in the OH group. Nutritional supplementation reduced waist circumference by approximately 7% in the OCN and OHN groups. In all groups, body weight increased progressively over time. At the end of the 12-week experimental period, the mean body weight in the OH group was 49.58 ± 4.05 g, representing an approximately 51% increase compared with the OC group. Supplementation with the nutritional formula reduced body weight in the OHN group to 41.34 ± 4.25 g, corresponding to an approximately 17% reduction compared with the OH group.

Food intake was recorded weekly at the cage level and expressed as estimated g/mouse/day. The estimated daily basal diet intake was 3.9, 3.8, 3.2, 3.4, and 3.0 g/mouse/day in the YC, OC, OCN, OH, and OHN groups, respectively. The specialized nutritional formula was administered by oral gavage at a fixed dose of 0.28 g/mouse, five times per week. For calculation of average daily energy intake, formula intake was averaged over seven days and corresponded to 0.83 kcal/mouse/day. Based on the caloric densities of the standard chow diet, HFD, and specialized multinutrient formula, the estimated total caloric exposure was 13.07, 12.73, 11.55, 17.34, and 16.13 kcal/mouse/day in the YC, OC, OCN, OH, and OHN groups, respectively. Within the standard-diet groups, OCN showed lower estimated caloric exposure than OC but did not show deterioration in muscle-related outcomes. Within the HFD-fed groups, OHN also showed lower estimated caloric exposure than OH. Therefore, body weight and adiposity-related outcomes were interpreted together with muscle histology and functional assessments.

Analysis of relative tissue weights normalized to body weight is shown in Table 1. Relative quadriceps weight was lower in the OC and OH groups than in the YC group. The OH group also showed higher relative adipose tissue weights than the OC group, including abdominal, epididymal, and subcutaneous fat depots. After nutritional supplementation, the most evident reduction was observed in abdominal fat weight in the OHN group, which decreased from 54.0 ± 5.3 mg/g body weight in the OH group to 29.0 ± 2.8 mg/g body weight in the OHN group. Because tissue-weight interpretation may be influenced by differences in body weight, relative tissue weights were interpreted together with the absolute tissue weights provided in Supplementary Table S3 and subsequent histological analyses.

### 3.2. Assessment of Skeletal Muscle Structural Damage and Lipid Accumulation

**Figure 2:**
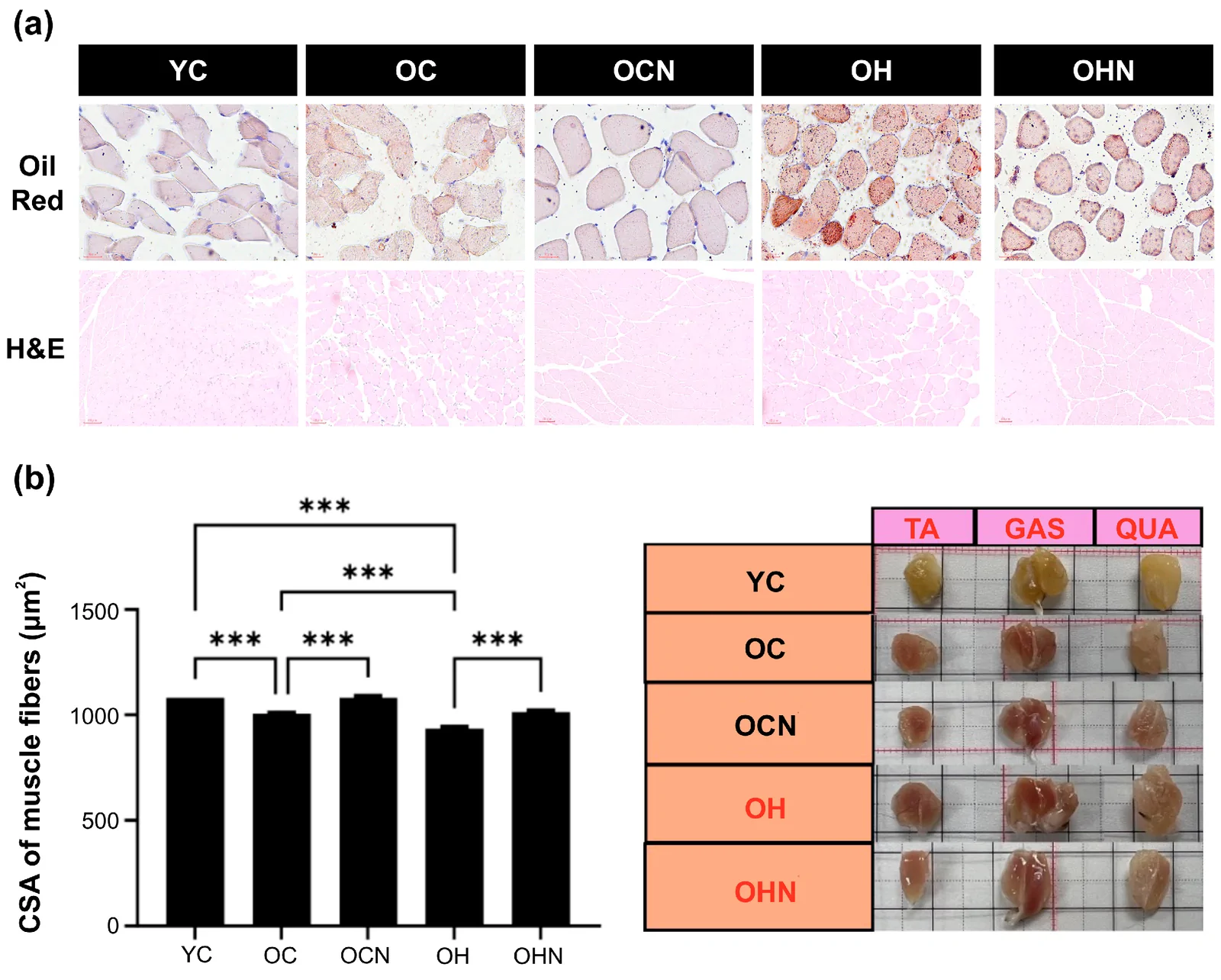
Effects of the specialized multinutrient formula on skeletal muscle morphology and intramuscular lipid deposition. (**a**) Representative Oil Red O-stained gastrocnemius muscle sections (scale bar = 30 μm) and H&E-stained sections (scale bar = 100 μm). Oil Red O staining was used for qualitative assessment only. (**b**) Quantitative analysis of muscle fiber cross-sectional area and representative skeletal muscle morphology. For qualitative presentation, one representative image was selected from each group. For quantitative CSA analysis, one image was analyzed for each mouse, and three mice were analyzed per group. Each mouse was treated as the biological experimental unit for statistical analysis. Data are presented as the mean ± SD; *n* = 3 mice/group for CSA analysis. Statistical significance was determined by one-way ANOVA followed by Tukey’s multiple-comparison test. *** *p* < 0.001. YC, young control; OC, adult control; OCN, adult control with nutritional formula; OH, adult high-fat diet; OHN, adult high-fat diet with nutritional formula.

Skeletal muscle morphology and lipid deposition were assessed by histological analyses (Figure 2). H&E staining revealed more muscle fiber disruption, increased vacuolization, and more disorganized fiber arrangement in the OC and OH groups compared with the YC group. These alterations were more evident in the OH group. In contrast, muscle fibers in the OCN and OHN groups showed a more compact organization and fewer vacuolar spaces than their corresponding non-supplemented groups.

The muscle fiber cross-sectional area (CSA) was reduced in both the OC and OH groups compared with the YC group, with the OH group showing a further decrease relative to the OC group. Supplementation with the specialized multinutrient formula increased CSA in both the OCN and OHN groups compared with their corresponding non-supplemented groups (Figure 2b).

Oil Red O staining was used as a qualitative assessment of intramuscular lipid deposition. Minimal lipid staining was observed in the YC group, whereas lipid deposition appeared more evident in the OC and OH groups, particularly in the OH group. Nutritional supplementation was associated with less extensive lipid staining in the OHN group compared with the OH group. Because Oil Red O staining was not quantified, these observations were interpreted qualitatively.

### 3.3. Muscle Functional Performance

**Figure 3:**
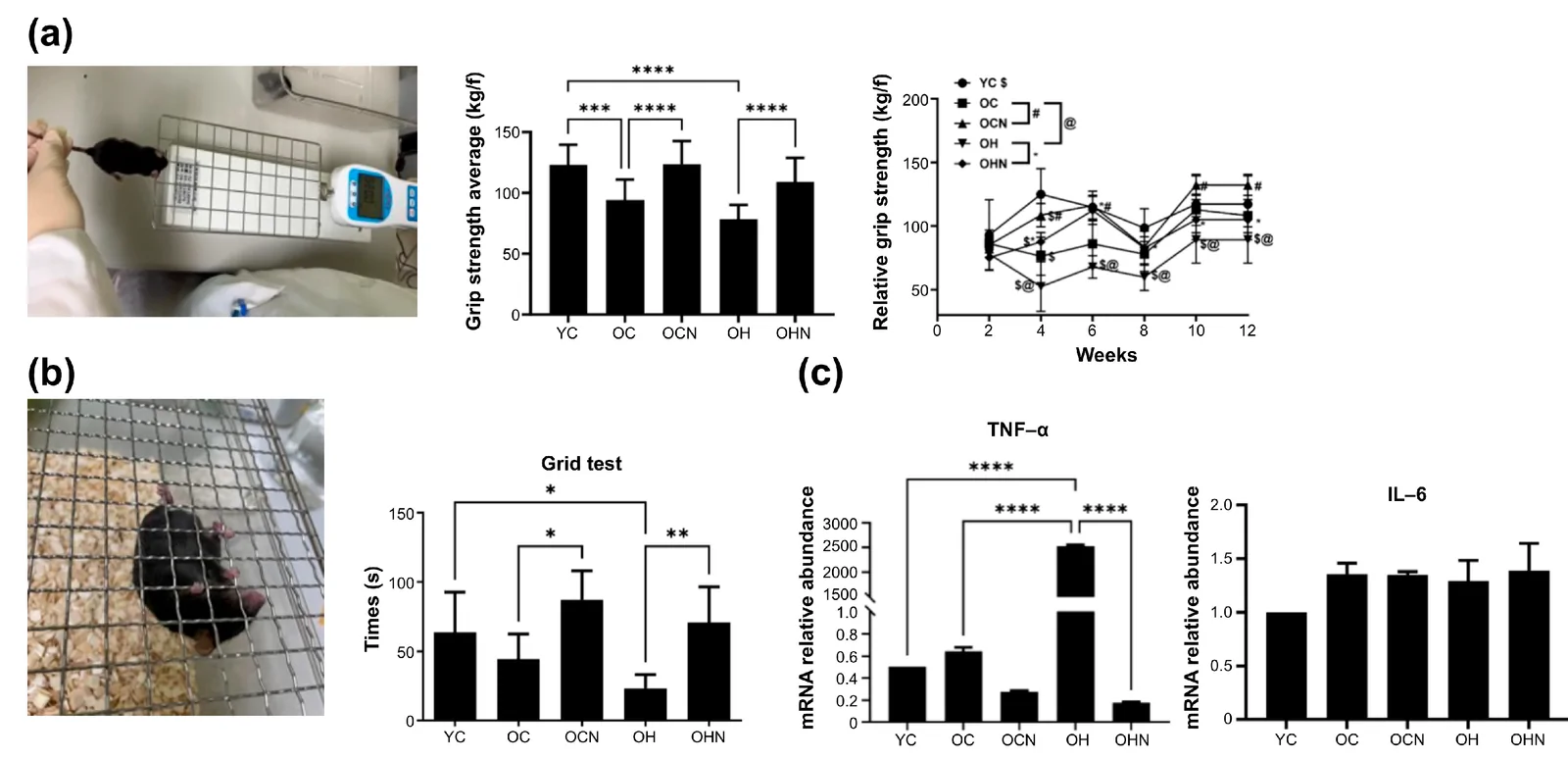
Effects of nutritional supplementation on muscle function and inflammatory gene expression. (**a**) Forelimb grip strength measured at weeks 2, 4, 6, 8, 10, and 12. Longitudinal grip strength data were analyzed using a mixed-effects model. (**b**) Grid hanging test assessing four-limb muscle endurance at week 12. (**c**) Relative mRNA expression levels of Tnf-α and Il-6 in gastrocnemius muscle determined by qRT-PCR. For qRT-PCR analysis, mouse-level ΔCt values were used for statistical analysis, and relative expression values are shown for presentation. Data are presented as the mean ± SD; *n* = 5 mice/group. Endpoint data were analyzed using one-way ANOVA followed by Tukey’s multiple-comparison test. For the longitudinal grip-strength plot in panel (**a**), $, #, @, and * indicate *p* < 0.05 versus YC, OC, OCN, and OH, respectively. For bracketed comparisons, *, **, ***, and **** indicate *p* < 0.05, *p* < 0.01, *p* < 0.001, and *p* < 0.0001, respectively. YC, young control; OC, adult control; OCN, adult control with nutritional formula; OH, adult high-fat diet; OHN, adult high-fat diet with nutritional formula.

Muscle function was assessed by the grip strength and grid hanging tests (Figure 3). Compared with the YC group, grip strength was reduced in the OC and OH groups, with the OH group showing the most pronounced reduction. In contrast, grip strength was improved in the OCN and OHN groups compared with their corresponding non-supplemented groups (Figure 3a).

Longitudinal analysis over the 12-week experimental period showed group- and time-dependent changes in grip strength from week 2 to week 12. The supplemented groups maintained higher grip strength values than their corresponding non-supplemented groups during the experimental period. Similar trends were observed in the grid hanging test, in which the OH group showed reduced hanging time, whereas the OCN and OHN groups showed improved hanging performance compared with the OC and OH groups, respectively (Figure 3b).

### 3.4. Analysis of Inflammatory Gene Expression

To evaluate the inflammatory status of skeletal muscle, mRNA expression levels of pro-inflammatory cytokines were analyzed by qRT-PCR (Figure 3c). In gastrocnemius muscle, Tnf-α mRNA expression was increased in the OH group compared with the YC and OC groups, reaching approximately 2500 arbitrary units. In the OHN group, this increase was reduced following nutritional supplementation. In contrast, Il-6 expression did not differ significantly among groups. These findings indicate that the inflammatory gene expression response was more evident for Tnf-α than for Il-6 under the present experimental conditions.

### 3.5. Analysis of Selected Apoptosis-, Proteolysis-, and Tissue Remodeling-Related Protein Markers

**Figure 4:**
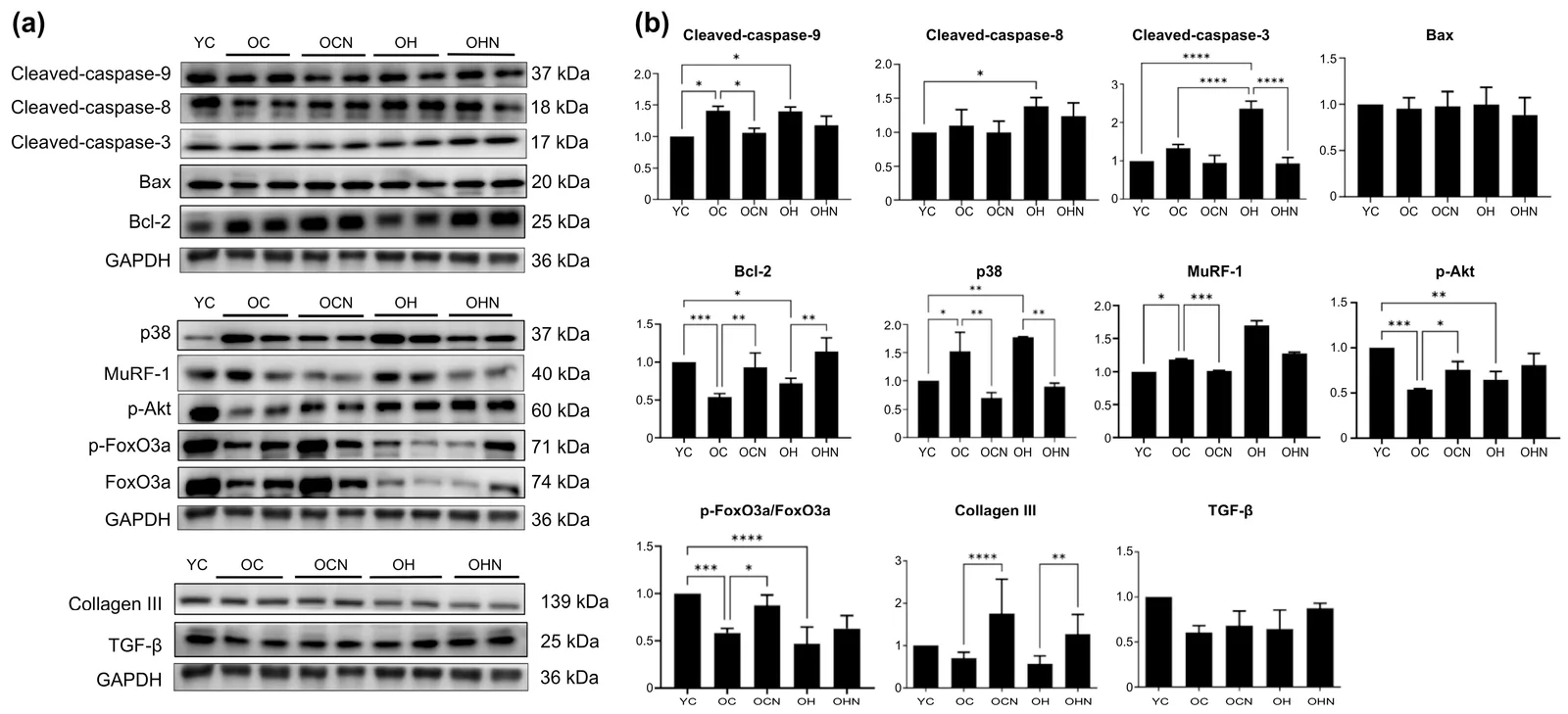
Assessment of selected apoptosis-, proteolysis-, and tissue remodeling-related protein markers in skeletal muscle by Western blot. (**a**) Representative Western blot images of gastrocnemius muscle. (**b**) Quantitative analysis of cleaved caspase-9, cleaved caspase-8, cleaved caspase-3, Bax, Bcl-2, p38 MAPK, MuRF-1, p-Akt, p-FoxO3a/FoxO3a ratio, collagen type III, and TGF-β. Data are presented as the mean ± SD; *n* = 5 mice/group. Statistical significance was determined by one-way ANOVA followed by Tukey’s multiple-comparison test. Because total p38 MAPK rather than phosphorylated p38 MAPK was measured, p38 MAPK was interpreted as total p38 MAPK protein expression. Because p-Akt was measured without total Akt normalization, p-Akt was interpreted as p-Akt abundance. * *p* < 0.05, ** *p* < 0.01, *** *p* < 0.001, and **** *p* < 0.0001. YC, young control; OC, adult control; OCN, adult control with nutritional formula; OH, adult high-fat diet; OHN, adult high-fat diet with nutritional formula.

Expression of proteins associated with caspase-related apoptosis, proteolysis-related signaling, and tissue remodeling was examined by Western blotting (Figure 4). Compared with the YC group, cleaved caspase-9 abundance was higher in both the OC and OH groups. The OH group also showed higher cleaved caspase-8 and cleaved caspase-3 abundance. These increases were reduced in the OCN and OHN groups following nutritional supplementation. Anti-apoptotic Bcl-2 abundance was lower in the OC and OH groups but higher in both the OCN and OHN groups (Figure 4b).

Among the examined proteolysis-related markers, the OC and OH groups showed increased p38 MAPK and MuRF-1 expression and a decreased p-FoxO3a/FoxO3a ratio. Dietary supplementation decreased p38 MAPK and MuRF-1 expression and increased p-Akt abundance and the p-FoxO3a/FoxO3a ratio in the OCN and OHN groups. Because total p38 MAPK rather than phosphorylated p38 MAPK was measured, these results were interpreted as changes in total p38 MAPK expression rather than direct evidence of p38 MAPK activation. Because p-Akt was measured without total Akt normalization, p-Akt was interpreted as p-Akt abundance rather than direct evidence of Akt pathway activity.

Proteins related to tissue remodeling were also examined. Compared with the YC group, the OC and OH groups showed lower collagen type III expression. Nutritional supplementation increased collagen type III expression in both the OCN and OHN groups. No clear group differences in transforming growth factor-β (TGF-β) expression were observed. Overall, these Western blot results were interpreted as changes in selected protein markers rather than direct evidence of complete pathway modulation.

## 4. Discussion

Sarcopenia and sarcopenic obesity are major geriatric syndromes characterized by progressive declines in skeletal muscle mass, strength, and functional performance, often accompanied by excessive adiposity and chronic low-grade inflammation. Previous studies have reported marked reductions in grip strength, muscle mass, and endurance in naturally aged male C57BL/6J mice aged 23–26 months, with a sarcopenia prevalence of 9–22% when classified using grip strength, muscle mass, and treadmill running time criteria [50]. These findings support the relevance of naturally aged C57BL/6J mice for modeling age-related declines in muscle mass and function. In the present study, 15-week-old mice were used as adult mice to evaluate muscle-related changes under standard-diet and HFD-fed conditions.

Natural aging models require extended experimental durations exceeding 18 months. For this reason, HFD feeding has been used to induce metabolic stress-related muscle alterations, as excessive lipid intake promotes chronic inflammation, mitochondrial dysfunction, impaired muscle remodeling, and intramuscular lipid infiltration [16,51,52]. In the present study, the combined use of adult mice and HFD feeding enabled evaluation of muscle dysfunction and lipid accumulation under adult HFD-associated metabolic stress.

Although the mice used in the present study had not reached an advanced natural age, the combination of adult physiological status and HFD feeding induced several sarcopenic obesity-related features, including impaired muscle function, increased adiposity, intramuscular lipid deposition, and alterations in selected inflammatory, cleaved caspase-, protein turnover-related, and remodeling markers. These pathological alterations were most evident in the OH group, supporting the use of this model for evaluating nutritional supplementation under adult HFD-associated muscle dysfunction and sarcopenic obesity-related metabolic stress.

Comparisons between OC and OCN and between OH and OHN were used to evaluate the association of nutritional supplementation with muscle-related outcomes under standard-diet and HFD-fed conditions. The OH group exhibited pronounced adverse changes, including increased body weight and waist circumference, excessive accumulation of abdominal and subcutaneous adipose tissues, reduced muscle fiber cross-sectional area, and marked impairments in grip strength and motor endurance. At the molecular level, increased abundance of cleaved caspase-3, caspase-8, and caspase-9, along with reduced abundance of the anti-apoptotic protein Bcl-2, suggested alterations in cleaved caspase-related markers under adult HFD-associated metabolic stress.

Importantly, estimated caloric exposure was calculated by incorporating dietary intake, the caloric density of each diet, and the caloric contribution of the gavaged nutritional formula. Estimated caloric exposure differed between supplemented and non-supplemented groups. Within the standard-diet groups, OCN showed lower estimated caloric exposure than OC, and within the HFD-fed groups, OHN showed lower estimated caloric exposure than OH. Therefore, differences in energy exposure may have contributed in part to body weight and adiposity-related outcomes. However, despite lower estimated caloric exposure, the supplemented groups did not show deterioration in muscle-related outcomes and instead showed improved grip strength, increased muscle fiber cross-sectional area, lower Tnf-α expression, and favorable changes in selected muscle-related protein markers. These findings suggest that caloric exposure alone may not fully explain the observed muscle-related outcomes, and that nutrient composition may also contribute to the effects of the formula. This distinction is particularly relevant in the context of sarcopenic obesity, where excessive caloric intake may coexist with inadequate micronutrient status and impaired muscle quality [43,44,45,46,47,48,49].

Supplementation with the specialized multinutrient formula resulted in consistent functional and structural improvements in both the OCN and OHN groups. In the OCN group, supplementation enhanced muscle strength, motor coordination, muscle fiber organization, and muscle fiber cross-sectional area under adult standard-diet conditions. These improvements were accompanied by increased p-Akt abundance and elevated p-FoxO3a/FoxO3a ratios, which suggested favorable changes in selected protein turnover-related markers. The OHN group similarly exhibited improvements in muscle performance and muscle fiber integrity, as well as less extensive intramuscular lipid staining. The nutritional intervention was also associated with lower Tnf-α expression, lower total p38 MAPK expression, and reduced cleaved caspase marker abundance under HFD-fed conditions.

Distinct phenotypic differences were evident between adult standard-diet and HFD-fed conditions. In the OC group, reduced relative quadriceps weight and reduced muscle fiber cross-sectional area were observed compared with the YC group. In contrast, the OH group exhibited greater body weight, waist circumference, and adipose tissue accumulation than did adult standard-diet controls, indicating an additional metabolic burden imposed by excessive dietary fat intake [1,53]. However, relative muscle weight alone cannot distinguish contractile muscle mass from intramuscular lipid infiltration. Therefore, these tissue-weight data were interpreted together with the absolute tissue weights provided in Supplementary Table S3 and histological findings. Accordingly, Oil Red O staining showed more evident intramuscular lipid deposition in the OH group, supporting the presence of myosteatosis-like changes and impaired muscle quality under HFD-fed conditions [16]. These findings underscore the importance of assessing both muscle quality and muscle mass in sarcopenic obesity.

The OH group exhibited substantially greater increases in body weight, waist circumference, and adipose tissue mass compared with adult standard-diet controls, highlighting the metabolic burden imposed by excessive dietary fat intake [14,54]. The observed changes in relative hindlimb muscle weight in the OH group may partly reflect differences in body weight and intramuscular lipid deposition, also known as myosteatosis, as supported by Oil Red O staining [16]. These findings further support the importance of assessing muscle quality rather than muscle mass alone in sarcopenic obesity-related metabolic stress.

Functional assessments further demonstrated that adult standard-diet and HFD-fed conditions were associated with declines in muscle strength and endurance, with the most severe impairments observed in the OH group. These results align with previous reports that chronic inflammation, proteolysis-related responses, and lipid accumulation are associated with skeletal muscle deterioration under aging and obesity-related conditions [55,56]. Our histological analyses corroborated the functional findings, revealing disrupted muscle fiber architecture, increased vacuolization, and reduced fiber cross-sectional area, particularly under HFD-fed conditions.

Notably, nutritional supplementation ameliorated muscle-related alterations in both adult standard-diet and HFD-fed mice. In the OCN group, supplementation was associated with improved muscle structural integrity and increased muscle fiber cross-sectional area. In the OHN group, supplementation improved body weight-related outcomes, waist circumference, muscle fiber organization, and functional performance while reducing abdominal fat accumulation and qualitative intramuscular lipid deposition based on Oil Red O staining.

In the HFD-fed model, supplementation did not clearly increase relative hindlimb muscle mass. Instead, the beneficial effects were primarily reflected in improved muscle quality, including increased muscle fiber cross-sectional area, less extensive intramuscular lipid deposition, and improved functional performance. These findings suggest that the nutritional formula may improve skeletal muscle quality primarily by limiting lipid accumulation and preserving muscle structure rather than by increasing gross muscle weight. These effects may be partly related to the combined nutrient composition of the multinutrient formula, including whey protein and branched-chain amino acids, which have been reported to affect lipid metabolism and inflammatory responses through AMPK activation, suppression of lipogenesis, and inhibition of adipocyte differentiation [35,38,57].

In the present study, improvements in grip strength were detectable as early as week 4 after initiating nutritional supplementation. This early functional response was observed within our experimental model and should therefore be interpreted as preclinical evidence. Indeed, previous clinical studies have reported that targeted multinutrient supplementation exerted beneficial effects on muscle-related outcomes among older adults after longer-term intervention [58]. β-glucan, a key polysaccharide component of the nutritional formula, has been shown to suppress FoxO3 activation and reduce expression of muscle-specific E3 ubiquitin ligases, such as MuRF-1 and Atrogin-1, thereby attenuating muscle atrophy [59]. These effects are consistent with our observations of lower MuRF-1 expression and a higher p-FoxO3a/FoxO3a ratio. In addition, β-glucan may influence host metabolic and inflammatory responses through modulation of gut microbiota. Because gut microbiota composition was not analyzed in the present study, this mechanism remains a possible explanation and requires further investigation.

To elucidate the molecular markers associated with the observed functional benefits, inflammatory gene expression was examined. Skeletal muscle from the OH group showed marked elevation of Tnf-α expression, which was reduced following nutritional supplementation. TNF-α is a central pro-inflammatory cytokine implicated in aging and metabolic disorders and is known to activate NF-κB and p38 MAPK signaling, thereby promoting muscle catabolism and apoptosis [17,60]. Lower Tnf-α expression may therefore represent one marker associated with the beneficial effects of nutritional supplementation. The absence of significant group differences in Il-6 expression reflects its complex, context-dependent role in skeletal muscle physiology. IL-6 may exert pro-inflammatory effects during chronic inflammation but serve anti-inflammatory or metabolic roles following acute exercise [61,62]. Although IL-6 has been implicated in early muscle wasting, particularly in synergy with TNF-α, its precise role in sarcopenia progression remains incompletely understood. These observations suggest that Tnf-α, rather than Il-6, was more responsive under the conditions of our experiments.

Consistent with increased inflammatory activity, cleaved caspase-related markers were higher in adult standard-diet and HFD-fed mice, as evidenced by increased abundance of cleaved caspase-9, caspase-8, and caspase-3. Higher cleaved caspase-8 abundance in the OH group suggested increased extrinsic apoptosis-related marker expression under HFD-associated metabolic and inflammatory stress [23,63,64]. Consistent with reports that nutritional interventions may stabilize mitochondrial function and affect apoptosis-related responses in aging tissues [65], nutritional supplementation reduced cleaved caspase-9 abundance in our mouse model. Because apoptosis rates were not directly measured, these findings should be interpreted as changes in cleaved caspase-related protein markers.

In the OH and OC groups, increased total p38 MAPK and MuRF-1 expression, together with lower p-Akt abundance and lower p-FoxO3a/FoxO3a ratios, indicated alterations in selected proteolysis-related markers under adult standard-diet and HFD-fed conditions. p38 MAPK is a key mediator of inflammation-induced muscle catabolism and promotes MuRF-1 expression, thereby accelerating myofibrillar protein degradation [17]. Increased p-Akt abundance and p-FoxO3a/FoxO3a ratio with nutritional supplementation suggested favorable changes in Akt/FoxO3a-related markers and MuRF-1 expression [20,66]. Because total p38 MAPK rather than phosphorylated p38 MAPK was measured, these data do not directly demonstrate p38 MAPK activation. In addition, because p-Akt was measured without total Akt normalization, these findings should be interpreted as p-Akt abundance rather than restored Akt activity.

Lastly, proteins involved in extracellular matrix remodeling and tissue repair were evaluated. Collagen type III expression was lower in skeletal muscle from the OC and OH groups, indicating altered extracellular matrix remodeling under adult standard-diet and HFD-fed conditions. This reduction may be partly explained by chronic low-grade inflammation and lipid-induced metabolic stress, which can disrupt extracellular matrix homeostasis and collagen synthesis during muscle repair. As a key component of the muscle extracellular matrix, collagen type III plays an essential role in muscle fiber stability and regeneration [67]. The ability of nutritional supplementation to increase collagen type III expression suggests favorable changes in the muscle repair microenvironment. In contrast, TGF-β expression did not differ significantly among groups. One possible explanation is that TGF-β activation may be more prominent during the acute phase of muscle injury and repair; however, the present study assessed skeletal muscle under chronic adult standard-diet and HFD-associated metabolic stress after 12 weeks of intervention. Therefore, endpoint total TGF-β expression may not fully reflect earlier, transient, or localized changes in TGF-β signaling activity [68,69,70].

Several limitations should be acknowledged. First, the sample size was relatively small. Second, the 15-week-old mice used in this study were adult mice; therefore, the findings should be interpreted as evidence from adult mice under standard-diet and HFD-associated metabolic stress conditions. Third, because the young mice were included as a reference group, the present design could not fully separate the independent effects of age, diet, and supplementation. Fourth, all groups underwent the same non-loaded ladder-climbing activity; therefore, the independent effect of exercise could not be separated from that of nutritional supplementation, and the findings should be interpreted under a standardized exercise background. Fifth, exercise sessions were performed during the daytime according to facility operation and animal handling schedules, which may represent a potential confounding factor because mice are nocturnal animals. Sixth, because food intake was recorded at the cage level and estimated caloric exposure differed among groups, body weight and adiposity-related outcomes should be interpreted cautiously. Seventh, differences in micronutrient composition between the standard chow and HFD may also have influenced the observed diet-related effects. Finally, because the formula contained multiple bioactive components, the contribution of each individual ingredient could not be determined through the current study design.

**Figure 5:**
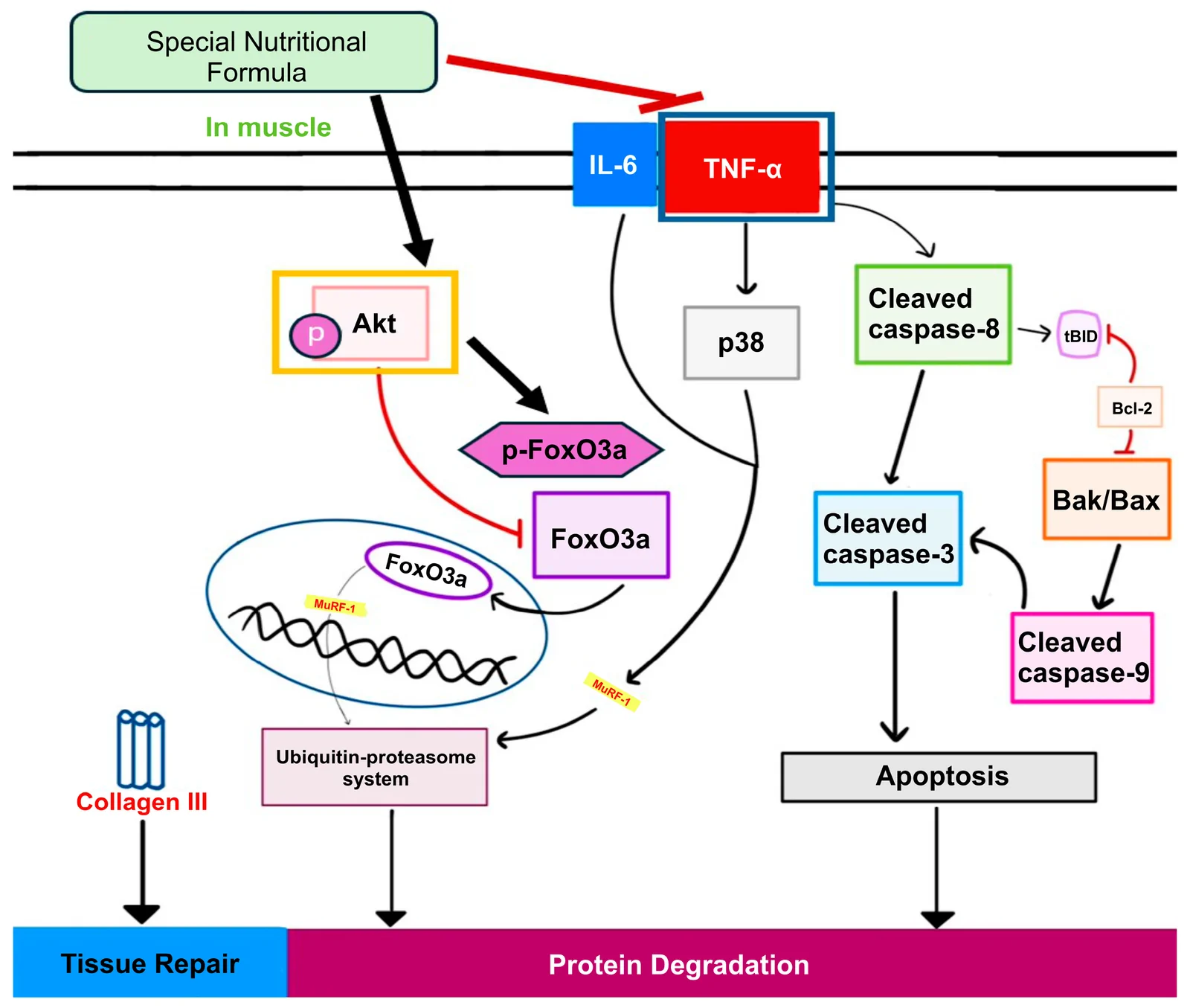
Proposed marker-based interpretation of molecular changes associated with nutritional supplementation in adult HFD-associated muscle dysfunction. Based on our experimental findings, adult standard-diet and HFD-fed conditions were associated with increased Tnf-α expression, higher cleaved caspase marker abundance, increased total p38 MAPK and MuRF-1 expression, lower p-Akt abundance, a lower p-FoxO3a/FoxO3a ratio, and lower collagen type III expression. Nutritional supplementation was associated with favorable changes in these selected markers. Because p38 MAPK phosphorylation, total Akt-normalized p-Akt expression, apoptosis rates, protein degradation rates, FoxO3a nuclear localization, and direct muscle regeneration were not measured, this schematic should be interpreted as a proposed marker-based framework rather than confirmation of complete pathway modulation.

Taken together, these findings suggest that the specialized multinutrient formula was associated with improved muscle function, muscle fiber morphology, and selected molecular markers under adult standard-diet and HFD-fed conditions. Because all experimental groups underwent the same ladder-climbing activity, the present findings should be interpreted under a standardized exercise background. Although estimated caloric exposure was lower in supplemented groups, muscle-related outcomes were maintained or improved, suggesting that caloric exposure alone may not fully explain the observed effects. The molecular results should be interpreted as changes in selected pathway-related markers rather than direct evidence of complete pathway modulation. These findings provide initial preclinical evidence that the specialized multinutrient formula may help attenuate HFD-associated muscle dysfunction and intramuscular lipid accumulation in adult mice. A schematic summary of these marker-based interpretations is provided in Figure 5.

## 5. Conclusions

The findings of the present study demonstrate that adult HFD-fed mice developed sarcopenic obesity-related features, including skeletal muscle structural alterations, functional decline, intramuscular lipid accumulation, and adipose tissue expansion. These alterations were accompanied by increased Tnf-α expression and changes in selected protein markers related to cleaved caspase-associated apoptosis, protein turnover, and tissue remodeling. Importantly, nutritional supplementation improved muscle functional performance and muscle fiber morphology, reduced intramuscular lipid deposition, and was associated with lower Tnf-α expression, reduced cleaved caspase marker abundance, lower total p38 MAPK and MuRF-1 expression, increased p-Akt abundance, a higher p-FoxO3a/FoxO3a ratio, and higher collagen type III expression. Although estimated caloric exposure was lower in supplemented groups, muscle-related outcomes were maintained or improved, suggesting that caloric exposure alone may not fully explain the observed effects. Collectively, these findings provide initial preclinical evidence that a specialized multinutrient nutritional strategy may help attenuate HFD-associated muscle dysfunction and intramuscular lipid accumulation in adult mice. Future randomized controlled trials in older adults with sarcopenic obesity are warranted to confirm these findings and establish optimal dosing strategies for clinical application.

## Figures and Tables

**Table 1:** Relative weights of hindlimb muscles and adipose tissues after 12 weeks.

**Group**	**Young Control (YC)**	**Adult Control (OC)**	**Adult Control + Nutritional Formula (OCN)**	**Adult HFD (OH)**	**Adult HFD + Nutritional Formula (OHN)**
	*n* = 5	*n* = 5	*n* = 5	*n* = 5	*n* = 5
Tibialis anterior (mg/g body weight)	2.4 ± 0.2 ^a^	2.8 ± 0.3 ^a^	3.2 ± 0.3 ^a^	2.3 ± 0.2 ^a^	2.3 ± 0.2 ^a^
Gastrocnemius (mg/g body weight)	6.8 ± 0.7 ^a^	7.2 ± 0.7 ^a^	7.0 ± 0.7 ^a^	5.7 ± 0.6 ^ab^	5.5 ± 0.5 ^b^
Quadriceps (mg/g body weight)	6.1 ± 0.6 ^a^	4.2 ± 0.4 ^bc^	5.7 ± 0.6 ^ac^	4.4 ± 0.4 ^b^	4.1 ± 0.4 ^b^
Abdominal fat (mg/g body weight)	4.0 ± 0.4 ^a^	9.0 ± 0.9 ^a^	5.0 ± 0.4 ^a^	54.0 ± 5.3 ^b^	29.0 ± 2.8 ^c^
Epididymal WAT (mg/g body weight)	16.0 ± 1.6 ^a^	19.0 ± 1.8 ^a^	13.0 ± 1.3 ^a^	56.0 ± 5.5 ^b^	59.0 ± 5.9 ^b^
Subcutaneous fat (mg/g body weight)	12.0 ± 1.2 ^a^	11.0 ± 1.1 ^a^	9.0 ± 0.9 ^a^	52.0 ± 5.2 ^b^	42.0 ± 4.2 ^b^

The weights of tibialis anterior, gastrocnemius, quadriceps, abdominal fat, epididymal white adipose tissue (WAT), and subcutaneous fat are expressed as milligrams per gram of body weight (mg/g), normalized to total body weight. Data are presented as the mean ± SD; *n* = 5 mice/group. Different superscript letters indicate significant differences among groups as determined by one-way ANOVA followed by Tukey’s multiple-comparison test (*p* < 0.05). Absolute tissue weights are provided in Supplementary Table S3. YC, young control; OC, adult control; OCN, adult control supplemented with the nutritional formula; OH, adult high-fat diet; OHN, adult high-fat diet supplemented with the nutritional formula.

## Data Availability

The datasets used and/or analyzed during the current study are available from the corresponding author upon reasonable request due to the institutional data management policy and the inclusion of unpublished supporting data.

## Supplementary Materials

The following supporting information can be downloaded at https://www.mdpi.com/article/10.3390/nu18172886/s1, Table S1. Available nutrient composition of the control diet, high-fat diet, and specialized nutritional formula per 100 g; Table S2. Estimated food intake and caloric exposure during the experimental period; Table S3. Absolute body, hindlimb muscle, and adipose tissue weights after 12 weeks.

## Abbreviations

The following abbreviations are used in this manuscript:

Akt/p-Akt	Protein kinase B/phosphorylated protein kinase B
ANOVA	Analysis of variance
AWGS	Asian Working Group for Sarcopenia
Bax	Bcl-2-associated X protein
BCA	Bicinchoninic acid
BCAAs	Branched-chain amino acids
Bcl-2	B-cell CLL/lymphoma 2
BCP	1-Bromo-3-chloropropane
BMI	Body mass index
cDNA	Complementary deoxyribonucleic acid
CSA	Cross-sectional area
DEPC	Diethyl pyrocarbonate
EASO	European Association for the Study of Obesity
ESPEN	European Society for Clinical Nutrition and Metabolism
FDA	Food and Drug Administration
FoxO3a/p-FoxO3a	Forkhead box O3a/phosphorylated forkhead box O3a
GAPDH	Glyceraldehyde-3-phosphate dehydrogenase
H&E	Hematoxylin and eosin
HFD	High-fat diet
IL-6	Interleukin-6
MAFbx	Muscle atrophy F-box (Atrogin-1)
MAPK	Mitogen-activated protein kinase
mTOR/mTORC1	Mammalian target of rapamycin (complex 1)
MuRF-1	Muscle RING finger-1
OC	Adult control
OCN	Adult control supplemented with multinutrient formula
OH	Adult high-fat diet
OHN	Adult high-fat diet supplemented with multinutrient formula
p38 MAPK	p38 mitogen-activated protein kinase
PGC-1α	Peroxisome proliferator-activated receptor gamma coactivator-1α
PVDF	Polyvinylidene difluoride
qRT-PCR	Quantitative real-time polymerase chain reaction
RIPA	Radioimmunoprecipitation assay
RNA	Ribonucleic acid
SD	Standard deviation
TA	Tibialis anterior
TGF-β	Transforming growth factor-beta
TNF-α	Tumor necrosis factor-alpha
UPS	Ubiquitin–proteasome system
WAT	White adipose tissue
YC	Young control

## References

[1] Larsson L., Degens H., Li M., Salviati L., Lee Y.I., Thompson W., Kirkland J.L., Sandri M. (2019). Sarcopenia: Aging-related loss of muscle mass and function. Physiol. Rev..

[2] Jung H.N., Jung C.H., Hwang Y.-C. (2023). Sarcopenia in youth. Metabolism.

[3] Hung W.-S., Chen Y.-J., Tsai T.-S., Lee C.-H., Fang J.-T., Wen M.-S., Lin C.-Y., Liao K.-C., Yen C.-L. (2026). Associations between the severity of sarcopenia and health-related quality of life in older adults. J. Clin. Med..

[4] Chen L.-K., Woo J., Assantachai P., Auyeung T.-W., Chou M.-Y., Iijima K., Jang H.C., Kang L., Kim M., Kim S. (2020). Asian Working Group for sarcopenia: 2019 consensus update on sarcopenia diagnosis and treatment. J. Am. Med. Dir. Assoc..

[5] Chien M.-Y., Huang T.-Y., Wu Y.-T. (2008). Prevalence of sarcopenia estimated using a bioelectrical impedance analysis prediction equation in community-dwelling elderly people in Taiwan. J. Am. Geriatr. Soc..

[6] Araújo L.P., Figueiredo Godoy A.C., Fortes Frota F., Barbalho Lamas C., Quesada K., Rucco Penteado Detregiachi C., Cressoni Araújo A., Miglino M.A., Landgraf Guiguer E., Santos de Argollo Haber R. (2025). Sarcopenia in the aging process: Pathophysiological mechanisms, clinical implications, and emerging therapeutic approaches. Int. J. Mol. Sci..

[7] Zhang S., Yang X., An N., Lv M., Yang L., Liu R., Hu S., Chen W., Feng W., Mao Y. (2025). Risk factors and predictive models for sarcopenia in older adults. Aging Med..

[8] Ji S., Jung H.-W., Baek J.Y., Jang I.-Y., Lee E. (2024). Sarcopenia as the mobility phenotype of aging: Clinical implications. J. Bone Metab..

[9] Jimenez-Gutierrez G.E., Martínez-Gómez L.E., Martínez-Armenta C., Pineda C., Martínez-Nava G.A., Lopez-Reyes A. (2022). Molecular mechanisms of inflammation in sarcopenia: Diagnosis and therapeutic update. Cells.

[10] Pinel S., Kelp N.Y., Bugeja J.M., Bolsterlee B., Hug F., Dick T.J.M. (2021). Quantity versus quality: Age-related differences in muscle volume, intramuscular fat, and mechanical properties in the triceps surae. Exp. Gerontol..

[11] Zhou Y., Su X., Tan H., Xiao J. (2025). Association between metabolic score for visceral fat index and bmi-adjusted skeletal muscle mass index in American adults. Lipids Health Dis..

[12] Gao Q., Mei F., Shang Y., Hu K., Chen F., Zhao L., Ma B. (2021). Global prevalence of sarcopenic obesity in older adults: A systematic review and meta-analysis. Clin. Nutr..

[13] Donini L.M., Busetto L., Bischoff S.C., Cederholm T., Ballesteros-Pomar M.D., Batsis J.A., Bauer J.M., Boirie Y., Cruz-Jentoft A.J., Dicker D. (2022). Definition and diagnostic criteria for sarcopenic obesity: ESPEN and EASO consensus statement. Obes. Facts.

[14] Batsis J.A., Villareal D.T. (2018). Sarcopenic obesity in older adults: Aetiology, epidemiology and treatment strategies. Nat. Rev. Endocrinol..

[15] Laurens C., Louche K., Sengenes C., Coué M., Langin D., Moro C., Bourlier V. (2016). Adipogenic Progenitors from obese human skeletal muscle give rise to functional white adipocytes that contribute to insulin resistance. Int. J. Obes..

[16] Wang L., Valencak T.G., Shan T. (2024). Fat infiltration in skeletal muscle: Influential triggers and regulatory mechanism. iScience.

[17] Brennan C.M., Emerson C.P., Owens J., Christoforou N. (2021). P38 mapks—Roles in skeletal muscle physiology, disease mechanisms, and as potential therapeutic targets. JCI Insight.

[18] Dalle S., Rossmeislova L., Koppo K. (2017). The role of inflammation in age-related sarcopenia. Front. Physiol..

[19] Bodine S.C., Baehr L.M. (2014). Skeletal muscle atrophy and the E3 ubiquitin ligases MuRF1 and MAFbx/atrogin-1. Am. J. Physiol. Endocrinol. Metab..

[20] Pang X., Zhang P., Chen X., Liu W. (2023). Ubiquitin-proteasome pathway in skeletal muscle atrophy. Front. Physiol..

[21] Kim S., You Y., Kim O.-K., Lee J., Chung J.W., Shim S., Kim K., Park J., Jun W. (2022). Silymarin Prevents high-fat diet-induced muscle atrophy by regulating protein degradation and synthesis in mice. J. Med. Food.

[22] Xia Q., Huang X., Huang J., Zheng Y., March M.E., Li J., Wei Y. (2021). The role of autophagy in skeletal muscle diseases. Front. Physiol..

[23] Dirks A., Leeuwenburgh C. (2002). Apoptosis in skeletal muscle with aging. Am. J. Physiol. Regul. Integr. Comp. Physiol..

[24] Bogdanis G.C., Giannaki C.D. (2023). Dietary supplements and musculoskeletal health and function. Nutrients.

[25] Chaudhuri R.H. (2024). The role of amino acids in skeletal muscle health and sarcopenia: A narrative review. J. Biomed. Res..

[26] Shin S. (2024). Association between dietary fiber intake and low muscle strength among Korean adults. Clin. Nutr. Res..

[27] Montiel-Rojas D., Nilsson A., Santoro A., Franceschi C., Bazzocchi A., Battista G., de Groot L.C.P.G.M., Feskens E.J.M., Berendsen A., Pietruszka B. (2020). Dietary fibre may mitigate sarcopenia risk: Findings from the NU-AGE cohort of older European adults. Nutrients.

[28] Corrochano A.R., Buckin V., Kelly P.M., Giblin L. (2018). Invited review: Whey proteins as antioxidants and promoters of cellular antioxidant pathways. J. Dairy Sci..

[29] Ariyoshi W., Hara S., Koga A., Nagai-Yoshioka Y., Yamasaki R. (2021). Biological effects of Β-glucans on osteoclastogenesis. Molecules.

[30] Kim H.J., Shin J.S., Kim W.G., Lee S.Y. (2023). Combined effect of polycan, a β-glucan from Aureobasidium pullulans, and regular resistance exercise on muscle strength, biomarkers, and fitness profiles in adults with relatively low skeletal muscle mass: A randomised controlled trial. Food Funct..

[31] Wang R., Wu X., Lin K., Guo S., Hou Y., Ma R., Wang Q., Wang R. (2022). Plasma metabolomics reveals Β-glucan improves muscle strength and exercise capacity in athletes. Metabolites.

[32] Rigamonti A.E., Leoncini R., Casnici C., Marelli O., De Col A., Tamini S., Lucchetti E., Cicolini S., Abbruzzese L., Cella S.G. (2019). Whey proteins reduce appetite, stimulate anorexigenic gastrointestinal peptides and improve glucometabolic homeostasis in young obese women. Nutrients.

[33] Lin C.-C., Shih M.-H., Chen C.-D., Yeh S.-L. (2021). Effects of adequate dietary protein with whey protein, leucine, and vitamin d supplementation on sarcopenia in older adults: An open-label, parallel-group study. Clin. Nutr..

[34] Katsanos C.S., Chinkes D.L., Paddon-Jones D., Zhang X.-J., Aarsland A., Wolfe R.R. (2008). Whey Protein ingestion in elderly persons results in greater muscle protein accrual than ingestion of its constituent essential amino acid content. Nutr. Res..

[35] West D., Abou Sawan S., Mazzulla M., Williamson E., Moore D. (2017). Whey protein supplementation enhances whole body protein metabolism and performance recovery after resistance exercise: A double-blind crossover study. Nutrients.

[36] Nowson C., O’Connell S. (2015). Protein requirements and recommendations for older people: A review. Nutrients.

[37] Weinert D.J. (2009). Nutrition and muscle protein synthesis: A descriptive review. J. Can. Chiropr. Assoc..

[38] Wolfe R.R. (2017). Branched-chain amino acids and muscle protein synthesis in humans: Myth or reality?. J. Int. Soc. Sports Nutr..

[39] Moriwaki M., Wakabayashi H., Sakata K., Domen K. (2019). The effect of branched chain amino acids-enriched nutritional supplements on activities of daily living and muscle mass in inpatients with gait impairments: A randomized controlled trial. J. Nutr. Health Aging.

[40] Deutz N.E.P., Bauer J.M., Barazzoni R., Biolo G., Boirie Y., Bosy-Westphal A., Cederholm T., Cruz-Jentoft A., Krznariç Z., Nair K.S. (2014). Protein intake and exercise for optimal muscle function with aging: Recommendations from the ESPEN expert group. Clin. Nutr..

[41] McColl T.J., Clarke D.C. (2024). Kinetic modeling of leucine-mediated signaling and protein metabolism in human skeletal muscle. iScience.

[42] Rennie M.J., Bohé J., Smith K., Wackerhage H., Greenhaff P. (2006). Branched-chain amino acids as fuels and anabolic signals in human muscle. J. Nutr..

[43] Binkley N.C., Suttie J.W. (1995). Vitamin K nutrition and osteoporosis. J. Nutr..

[44] Meacci E., Chirco A., Garcia-Gil M. (2024). Potential vitamin E Signaling mediators in skeletal muscle. Antioxidants.

[45] Gogulothu R., Nagar D., Gopalakrishnan S., Garlapati V.R., Kallamadi P.R., Ismail A. (2020). Disrupted expression of genes essential for skeletal muscle fibre integrity and energy metabolism in vitamin D deficient rats. J. Steroid Biochem. Mol. Biol..

[46] Wacker M., Holick M. (2013). Vitamin D—Effects on skeletal and extraskeletal health and the need for supplementation. Nutrients.

[47] Girgis C.M., Clifton-Bligh R.J., Hamrick M.W., Holick M.F., Gunton J.E. (2013). The roles of vitamin D in skeletal muscle: Form, function, and metabolism. Endocr. Rev..

[48] Wellmann K.B., Kim J., Urso P.M., Smith Z.K., Johnson B.J. (2021). Evaluation of vitamin A status on myogenic gene expression and muscle fiber characteristics. J. Anim. Sci..

[49] Alfaqih M., Tarawan V., Sylviana N., Goenawan H., Lesmana R., Susianti S. (2022). Effects of vitamin D on satellite cells: A systematic review of in vivo studies. Nutrients.

[50] Kerr H.L., Krumm K., Anderson B., Christiani A., Strait L., Li T., Irwin B., Jiang S., Rybachok A., Chen A. (2024). Mouse sarcopenia model reveals sex- and age-specific differences in skeletal muscle decline. J. Nutr. Biochem..

[51] Zhang Y., Luo C., Huang P., Cheng Y., Ma Y., Gao J., Ding H. (2025). Luteolin alleviates muscle atrophy, mitochondrial dysfunction and abnormal FNDC5 expression in high fat diet-induced obese rats and palmitic acid-treated C2C12 myotubes. J. Nutr. Biochem..

[52] Jin J.B., Robinson A., Soukup T., Black E., Abit A., Hammer S.M., Han A., Lucas E., Kim Y., Bae J. (2025). Metabolic and molecular regulation in skeletal muscle dysfunction and regeneration. Front. Cell Dev. Biol..

[53] Clark B.C., Manini T.M. (2008). Sarcopenia != dynapenia. J. Gerontol. A Biol. Sci. Med. Sci..

[54] Castillo Í.M.P., Argilés J.M., Rueda R., Ramírez M., Pedrosa J.M.L. (2025). Skeletal muscle atrophy and dysfunction in obesity and type-2 diabetes mellitus: Myocellular mechanisms involved. Rev. Endocr. Metab. Disord..

[55] Colleluori G., Villareal D.T. (2021). Aging, obesity, sarcopenia and the effect of diet and exercise intervention. Exp. Gerontol..

[56] Batsis J.A., Mackenzie T.A., Jones J.D., Lopez-Jimenez F., Bartels S.J. (2016). Sarcopenia, sarcopenic obesity and inflammation: Results from the 1999–2004 National Health and Nutrition Examination Survey. Clin. Nutr..

[57] Kjøbsted R., Hingst J.R., Fentz J., Foretz M., Sanz M.N., Pehmøller C., Shum M., Marette A., Mounier R., Treebak J.T. (2018). AMPK in skeletal muscle function and metabolism. FASEB J..

[58] Bauer J.M., Verlaan S., Bautmans I., Brandt K., Donini L.M., Maggio M., McMurdo M.E.T., Mets T., Seal C., Wijers S.L. (2015). Effects of a vitamin D and leucine-enriched whey protein nutritional supplement on measures of sarcopenia in older adults, the PROVIDE study: A randomized, double-blind, placebo-controlled trial. J. Am. Med. Dir. Assoc..

[59] Lim J., Lee Y.J., Cho H., Park D., Jung G., Ku S.K., Choi J. (2018). Extracellular polysaccharides purified from aureobasidium pullulans SM-2001 (polycan) inhibit dexamethasone-induced muscle atrophy in mice. Int. J. Mol. Med..

[60] Stratos I., Behrendt A.-K., Anselm C., Gonzalez A., Mittlmeier T., Vollmar B. (2022). Inhibition of Tnf-α restores muscle force, inhibits inflammation, and reduces apoptosis of traumatized skeletal muscles. Cells.

[61] Pedersen B.K., Febbraio M.A. (2008). Muscle as an endocrine organ: Focus on muscle-derived interleukin-6. Physiol. Rev..

[62] Nara H., Watanabe R. (2021). Anti-inflammatory effect of muscle-derived interleukin-6 and its involvement in lipid metabolism. Int. J. Mol. Sci..

[63] Wu J., Lin S., Chen W., Lian G., Wu W., Chen A., Sagor M.I.H., Luo L., Wang H., Xie L. (2023). TNF-α contributes to sarcopenia through caspase-8/caspase-3/GSDME-mediated pyroptosis. Cell Death Discov..

[64] Huang Y., Wang C., Cui H., Sun G., Qi X., Yao X. (2025). Mitochondrial dysfunction in age-related sarcopenia: Mechanistic insights, diagnostic advances, and therapeutic prospects. Front. Cell Dev. Biol..

[65] López-Domínguez J.A., Khraiwesh H., González-Reyes J.A., López-Lluch G., Navas P., Ramsey J.J., de Cabo R., Burón M.I., Villalba J.M. (2013). Dietary fat modifies mitochondrial and plasma membrane apoptotic signaling in skeletal muscle of calorie-restricted mice. Age.

[66] Jaiswal N., Gavin M., Loro E., Sostre-Colón J., Roberson P.A., Uehara K., Rivera-Fuentes N., Neinast M., Arany Z., Kimball S.R. (2022). AKT controls protein synthesis and oxidative metabolism via combined Mtorc1 and FOXO1 signalling to govern muscle physiology. J. Cachexia Sarcopenia Muscle.

[67] Ahmad K., Shaikh S., Chun H.J., Ali S., Lim J.H., Ahmad S.S., Lee E.J., Choi I. (2023). Extracellular matrix: The critical contributor to skeletal muscle regeneration—A comprehensive review. Inflamm. Regen..

[68] Delaney K., Kasprzycka P., Ciemerych M.A., Zimowska M. (2017). The role of Tgf-β1 during skeletal muscle regeneration. Cell. Biol. Int..

[69] Girardi F., Taleb A., Ebrahimi M., Datye A., Gamage D.G., Peccate C., Giordani L., Millay D.P., Gilbert P.M., Cadot B. (2021). TGFβ signaling curbs cell fusion and muscle regeneration. Nat. Commun..

[70] Ismaeel A., Kim J.-S., Kirk J.S., Smith R.S., Bohannon W.T., Koutakis P. (2019). Role of transforming growth factor-β in skeletal muscle fibrosis: A review. Int. J. Mol. Sci..

