# Supplementary material for: A Specialized Multinutrient Formula Attenuates High-Fat-Diet-Associated Muscle Dysfunction and Sarcopenic Obesity-Related Features in Adult Mice
Source: Nutrients. 2026 Sep 3;18(17):2886. doi: 10.3390/nu18172886 (PMC13567642; doi:10.3390/nu18172886)
Supplement: Supplementary file 1 [file nutrients-18-02886-s001.zip › nutrients-4495310-supplementary.pdf]

Supplementary Table S1. Available nutrient composition of the control diet, high-fat diet, and specialized nutritional formula per 100 g.

**Nutrient content**	**Control diet (100g)**	**High fat diet (100g)**	**Special nutritional formula (100g)**
Calories (kcal)	335	510	413
Protein (g)	24.1	23.1	22.2
Fat (g)	5.1	34.9	9.2
Saturated fatty acid (g)	1.39	13.68	1.6
Carbohydrates (g)	48.1	25.9	61.6
Dietary Fiber (g)	5.3	6.5	2.5
Minerals			
Fe (mg)	24	6.4	6
Se (μg)	41	29	30
Vitamins			
Vitamin A (IU)	1800	520	1733
Vitamin D (IU)	460	130	320
Vitamin E (IU)	4.2	6.72	14.9
Vitamin K (μg)	130	65	35
Vitamin C (mg)	0	0	166
Added BCAAs (Branched-Chain Amino Acids) (mg)	0	0	4521
Added β-Glucan (mg)	0	0	33.06

Values are presented per 100 g. Nutrient values were compiled from available diet composition information and the product nutrition label. Added BCAAs and added β-glucan indicate additional functional ingredients rather than total amino acid or polysaccharide contents. Therefore, the value of 0 for the control diet and high-fat diet indicates that these components were not added as isolated functional ingredients; it does not indicate absence of amino acids or polysaccharides in the diets.

Supplementary Table S2. Estimated food intake and caloric exposure during the experimental period.

Group	Animals per group	Basal diet	Mean basal diet intake (g/mouse/day)	Basal diet energy (kcal/mouse/day)	Formula administration	Total energy intake (kcal/mouse/day)
YC	5	LabDiet 5001	3.9	13.07	Water	**13.07**
OC	5	LabDiet 5001	3.8	12.73	Water	**12.73**
OCN	5	LabDiet 5001	3.2	10.72	0.28 g/mouse, 5×/week	**11.55**
OH	5	TestDiet 58Y1	3.4	17.34	Water	**17.34**
OHN	5	TestDiet 58Y1	3.0	15.30	0.28 g/mouse, 5×/week	**16.13**

The specialized nutritional formula was administered by oral gavage at a fixed dose of 0.28 g per mouse, five times per week. For calculation of average daily energy intake, formula intake was averaged over seven days (0.28 × 5/7 = 0.20 g/mouse/day), corresponding to 0.83 kcal/mouse/day. Control mice received an equivalent volume of water by oral gavage.

Supplementary Table S3. Absolute body, hindlimb muscle, and adipose tissue weights after 12 weeks.

**Parameter**	**YC**	**OC**	**OCN**	**OH**	**OHN**
Body weight (g)	28.74 ± 1.59	32.88 ± 1.25	29.42 ± 0.74	49.58 ± 4.05	41.34 ± 4.25
Tibialis anterior (g)	0.07 ± 0.01	0.09 ± 0.01	0.09 ± 0.02	0.11 ± 0.02	0.09 ± 0.02
Gastrocnemius (g)	0.19 ± 0.02	0.24 ± 0.03	0.21 ± 0.01	0.28 ± 0.04	0.23 ± 0.02
Quadriceps (g)	0.17 ± 0.02	0.14 ± 0.03	0.17 ± 0.02	0.22 ± 0.02	0.17 ± 0.03
Abdominal fat (g)	0.11 ± 0.01	0.30 ± 0.14	0.15 ± 0.07	2.71 ± 0.89	1.20 ± 0.13
Epididymal WAT (g)	0.46 ± 0.07	0.63 ± 0.04	0.38 ± 0.06	2.75 ± 0.45	2.46 ± 0.43
Subcutaneous fat (g)	0.34 ± 0.08	0.36 ± 0.04	0.26 ± 0.04	2.58 ± 0.66	1.75 ± 0.34

Data are presented as the mean ± SD; n = 5 mice/group. YC, young control; OC, adult control; OCN, adult control supplemented with the nutritional formula; OH, adult high-fat diet; OHN, adult high-fat diet supplemented with the nutritional formula; WAT, white adipose tissue.
